# Gestational Diabetes Mellitus: Clinical Characteristics and Perinatal Outcomes in a Multiethnic Population of North Italy

**DOI:** 10.1155/2021/9474805

**Published:** 2021-12-26

**Authors:** M. Caputo, V. Bullara, C. Mele, M. T. Samà, M. Zavattaro, A. Ferrero, T. Daffara, I. Leone, G. Giachetti, V. Antoniotti, D. Longo, A. De Pedrini, P. Marzullo, V. Remorgida, F. Prodam, G. Aimaretti

**Affiliations:** ^1^Endocrinology, Department of Translational Medicine, Università del Piemonte Orientale, Novara, Italy; ^2^Department of Health Sciences, Università del Piemonte Orientale, Novara, Italy; ^3^SCDU Endocrinologia, AOU “Maggiore della Carità” Novara, Novara, Italy; ^4^Gynecology and Obstetrics, Department of Translational Medicine, Università del Piemonte Orientale, Novara, Italy; ^5^IRCCS Istituto Auxologico Italiano, Laboratory of Metabolic Research, Novara, Italy

## Abstract

**Aim:**

To evaluate clinical characteristics and perinatal outcomes in a heterogeneous population of Caucasians born in Italy and High Migration Pressure Countries (HMPC) women with GDM living in Piedmont, North Italy.

**Methods:**

We retrospectively analyzed data from 586 women referring to our unit (2015–2020). Epidemiological (age and country of origin) and clinical-metabolic features (height, weight, family history of DM, parity, previous history of GDM, OGTT results, and GDM treatment) were collected. The database of certificates of care at delivery was consulted in relation to neonatal/maternal complications (rates of caesarean sections, APGAR score, fetal malformations, and neonatal anthropometry).

**Results:**

43.2% of women came from HMPC; they were younger (*p* < 0.0001) and required insulin treatment more frequently than Caucasian women born in Italy (*χ*^2^ = 17.8, *p*=0.007). Higher fasting and 120-minute OGTT levels and gestational BMI increased the risk of insulin treatment (OGTT T0: OR = 1.04, CI 95% 1.016–1.060, *p*=0.005; OGTT T120: OR = 1.01, CI 95% 1.002–1.020, *p*=0.02; BMI: OR = 1.089, CI 95% 1.051–1.129, *p* < 0.0001). Moreover, two or more diagnostic OGTT glucose levels doubled the risk of insulin therapy (OR = 2.03, IC 95% 1.145–3.612, *p*=0.016). We did not find any association between ethnicities and neonatal/maternal complications.

**Conclusions:**

In our multiethnic GDM population, the need for intensive care and insulin treatment is high in HPMC women although the frequency of adverse peripartum and newborn outcomes does not vary among ethnic groups. The need for insulin therapy should be related to different genetic backgrounds, dietary habits, and Nutrition Transition phenomena. Thus, nutritional intervention and insulin treatment need to be tailored.

## 1. Introduction

Gestational diabetes mellitus (GDM) is the most common endocrinological disorder during pregnancy, with a prevalence of 4–12% [1]. According to the most recent classification, GDM has been defined as a condition of hyperglycemia occurring in the second or third trimester of pregnancy after excluding pregestational diabetes mellitus [2].

In most cases, the pathogenesis of GDM is related to a mother's relative beta-cell failure in respect to the pregnancy insulin resistance. Physiologically, the increase of placental hormones antagonizes insulin action in the second and third trimesters of pregnancy, leading to insulin resistance. This mechanism provides glucose to the fetus, and a gradual increase in insulin secretion is needed to maintain blood glucose in the normal range also in the pregnant woman [3, 4]. Because women with GDM have a blunted suppression of the endogenous glucose production that results into a postabsorptive hyperglycemia, the 75 g oral glucose tolerance test (OGTT) is the gold standard for the diagnosis [2].

Uncontrolled maternal hyperglycemia causes several potential short-term and long-term metabolic and clinical complications in the dyad [5]. The landmark 2008 HAPO study [6] showed that maternal hyperglycemia increases the risk of preeclampsia, preterm delivery, caesarean section delivery, macrosomic infants, shoulder dystocia, hypoglycemia, and neonatal hyperbilirubinemia. Then, the HAPO follow-up study demonstrated that GDM is associated with a long-term risk of type 2 diabetes mellitus (T2DM) in the mother and obesity in the offspring likely due to transgenerational epigenetic effects [7].

The prevalence of GDM is estimated to be globally growing and reflects the increase of obesity and unhealthy lifestyle habits and other environmental factors among women of reproductive age [8, 9].

Furthermore, advanced maternal age, family history of T2DM, previous GDM, multiparity, genetic factors, polycystic ovarian syndrome, and smoking are all further risk factors for GDM [10, 11]. Interestingly, the prevalence of GDM diverges among the ethnic groups likely because of multifactorial reasons, including genetic factors, body fat composition, and lifestyle with a transition to worse nutrition habits than in origin countries with foods rich in saturated fats and sugars. Women from Southeast Asia, the Eastern Pacific region, and North Africa seem to be particularly predisposed to GDM due to both genetic variants associated with insulin resistance and epigenetic adaptation to *in utero* nutritional restriction, in which environmental pressure plays a critical role [12].

Our study aims to evaluate clinical characteristics and perinatal outcomes of women affected by GDM referring to a single Italian Diabetes Centre and to consider differences between Caucasian women who were born in Italy and those coming from High Migration Pressure Countries (HMPC).

## 2. Patients and Methods

### 2.1. Patients

We retrospectively analyzed data regarding patients affected by GDM referred to our Diabetes Centre (University Hospital “Maggiore della Carità,” Novara, Italy) in the period encompassed from the 1^st^ January 2015 to 31^st^ December 2020. We included patients who underwent the OGTT for GDM according to current guidelines and who exhibited at least one blood glucose concentration exceeding normal pregnancy-related cutoffs at fasting, 1-hour, or 2-hour (92, 153, 180 mg/dl, respectively) [2].

Patients diagnosed with pregestational diabetes mellitus or any form of diabetes other than GDM were excluded from the study.

This study was conducted following the 1964 Declaration of Helsinki and its later amendments.

### 2.2. Standard of Care

On their first appointment and during the follow-up after GDM diagnosis, all pregnant women received a multidisciplinary visit in the Diabetes Centre from a diabetologist, a certified dietician, and a nurse. Patients were informed about maternal and fetal risks related to untreated GDM. A diet according to BMI range before pregnancy, basal metabolism, and adjusted for trimester relating to protein amount was given to them. An education to self-monitor blood glucose at fasting and 1 h after breakfast, lunch, and dinner was performed. Insulin treatment was started if patients did not meet the ADA glycemic goals [2]. The clinical follow-up was scheduled every 2-3 weeks according to glucose level ranges at fasting and one hour after meals.

### 2.3. Data Collection

By reviewing the clinical records, we collected the following data:

- Epidemiological information (age at diagnosis and country of origin)
- Past medical history and clinical information (height, weight, BMI, family history of DM, parity, and previous history of GDM)
- Metabolic features (OGTT results and GDM treatment)
- Type of treatment (diet or insulin)

**Figure 1:**
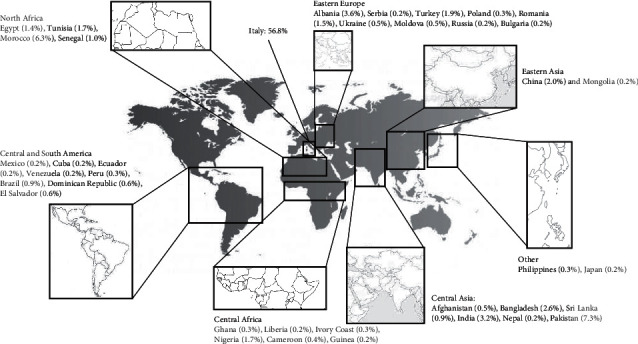
Macrogeographical areas of origin of pregnant women affected by GDM.

Apart from Caucasians born in Italy women, maternal ethnicity was self-reported by subjects and, considering the different countries of origin, the following macrogeographical areas were categorized, as represented in Figure 1: Eastern Europe, Eastern Asia, North Africa, Central Africa, Central and South America, and others.

The database of certificates of care at delivery (CedAP: Certificato di Assistenza al parto) has been inquired for information on neonatal/maternal complications [13].

The assessed parameters are listed in Table 1.

Anthropometric values of newborns were classified as AGA (Adequate for Gestational Age), SGA (Small for Gestational Age), and LGA (Large for gestational Age). LGA was defined as a weight greater than the 90^th^ percentile and SGA as less than the 10^th^ percentile [14].

### 2.4. Statistical Analysis

Statistical analyses were performed using SPSS version 26.0 (IBM Corp., Armonk, NY) on log-transformed data to correct the non-Gaussian distribution obtained by the Shapiro–Wilk test. In the text and tables, values are expressed as mean ± standard deviation (SD) or absolute number and percentage. Univariate ANOVA and Chi-square tests were used for comparison between groups. Pearson's correlation analysis was applied to identify significant associations between continuous variables. Multivariable regression analysis was conducted to identify predictors of OGTT glucose levels, APGAR score, and newborn anthropometric parameters. The multilinear model for OGTT glucose levels included age at diagnosis, BMI, geographic origin as described above, GDM in previous pregnancies, and family history. The multilinear model for APGAR score included age at diagnosis, geographic origin, BMI at the time of the first visit, type of GDM treatment (diet or insulin), type of delivery, and fetal presentation. The multilinear model for each anthropometric parameter (weight, height, and head circumference) included age at diagnosis, BMI at the time of the first visit, geographic origin, GDM in previous pregnancies, gestational age, and type of treatment. *β*-Coefficients and significance values obtained from the models were reported. Multinomial logistic regression analysis was performed to test the effect of OGTT glucose levels and maternal BMI (independent variables) on the treatment choice (dependent variable; insulin = 0, diet = 1). CI 95% was reported. Statistical significance was set at 5%.

## 3. Results

### 3.1. Baseline Characteristics

#### 3.1.1. Epidemiology

In the study period, a total of 586 patients were referred to our Centre for GDM.

The 56.8% of women were Italian-born Caucasians, while 43.2% came from HMPC: Asia 17.6%, Africa 13.5%, Eastern Europe 8.9%, and South America 3.2%.

**Figure 2:**
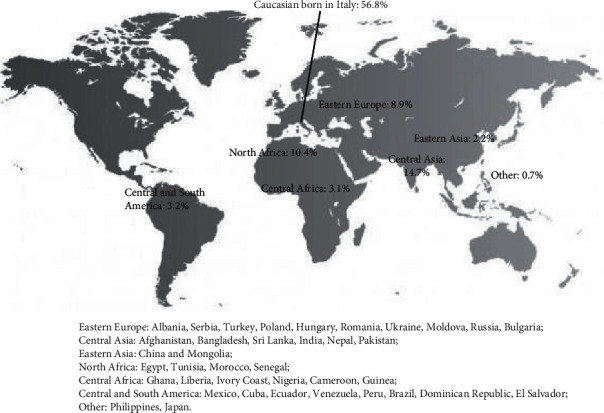
Distribution of pregnant women affected by GDM according to macrogeographical areas of origin.

Considering the macrogeographical areas of origin, the distribution of pregnant women was as follows (Figure 2):

- Central Asia: 14.7% (*N* = 86)
- North Africa: 10.4% (*N* = 61)
- Eastern Europe: 8.9% (*N* = 52)
- Central and South America: 3.2% (*N* = 19)
- Central Africa: 3.1% (*N* = 18)
- Eastern Asia: 2.2% (*N* = 13)
- Other: 0.7% (*N* = 4)

#### 3.1.2. Risk Factors

Information about GDM risk factors and differences among groups according to geographic and ethnic origin are reported in Table 2.

The age at diagnosis was 34.2 ± 5.0 years with differences between ethnic groups, as immigrant women were younger than Caucasians born in Italy (*p* < 0.0001). At the first visit, BMI was 29.1 ± 5.5 kg/m^2^. More than half of patients (60.4%) had a family history of diabetes mellitus among first-degree relatives (Table 2).

Considering parity, 33.6% of women were at the first pregnancy, 39.2% the second, 18.1% the third, and the remaining 9.1% had more than three pregnancies. The number of pregnancies differed between ethnic groups (*p* < 0.0001), with more in HMPC than Caucasian women born in Italy.

Most of the patients (85.5%) had a negative history of spontaneous abortions, 8.7% reported one miscarriage, and the remaining 5.8% reported multiple abortions, without differences between ethnic groups.

Considering nonprimiparous subjects (*N* = 386), most of them (68.9%) had no previous GDM; of 120 patients (31.1%) with previous GDM, 14 had it in two or more pregnancies.

### 3.2. Gestational Metabolic Characteristics

Information about gestational metabolic characteristics and differences among groups according to geographic and ethnic origin are reported in Table 3.

34.1% of patients had fasting blood glucose levels above cutoff values, 13.4% had impaired glucose levels at 60 minutes, and 10.4% at 120 minutes after OGTT. The 42.1% of patients had more than one value above the cutoff.

Central African and central Asian women had lower 60 min blood glucose levels after OGTT than Caucasian women born in Italy (Central African: 150.5 ± 41.1, Central Asian: 160.1 ± 36.8 vs. 170.8 ± 34.9 mg/dl, *p* < 0.05 for both).

Moreover, the prevalence of women undergoing insulin therapy was different among groups: HPMC women required more frequently than inborn counterpart insulin treatment (*χ*^2^ = 17.8, *p*=0.007).

A multivariable regression analysis was conducted to identify potential independent predictors of OGTT glucose levels. The multilinear model for each OGTT point included age at diagnosis, BMI at the time of the first visit, geographic origin, previous pregnancies with GDM, and family history of diabetes. While we did not identify independent predictors for fasting glucose levels, 60-minute glucose OGTT levels were independently predicted by ethnicity (*B* = 3.003, CI 95% 0.095–5.910, *p*=0.043) and 120-minute blood glucose OGTT levels by age (*B* = 1.487, CI 95% 0.661–2.313, *p* < 0.0001) and ethnicity (*B* = 2.738, CI 95% 0.257–5.220, *p*=0.03).

Higher fasting and 120-minute OGTT levels, as well as BMI at the time of the first visit, increased the risk of insulin treatment (OGTT T0: OR = 1.04, CI 95% 1.016–1.060, *p*=0.005; OGTT T120: OR = 1.01, CI 95% 1.002–1.020, *p*=0.02; BMI: OR = 1.089, CI 95% 1.051–1.129, *p* < 0.0001). Moreover, the presence of two or more diagnostic OGTT glucose levels doubled the risk of starting insulin therapy (OR = 2.03, IC 95% 1.145–3.612, *p*=0.016).

### 3.3. Peripartum and Newborn Characteristics

Peripartum and newborn characteristics and differences among groups according to the area of origin are reported in Table 4.

The delivery was vaginal for most patients (67.4%), while 32.6% underwent caesarean section (as an elective and emergency procedure in 17.2% and 15.4% of cases, respectively).

Gestational age at delivery was 38.4 ± 1.4 weeks.

One- and five-minute APGAR score ranged between 7 and 10 points for most of the patients (92.7% and 98.2%, respectively).

The frequency of newborn malformations was 1.2%, and shoulder dystocia was reported in 2 Central Asian women (0.6%). A stillbirth was detected (0.2%) in the Asian cohort.

Considering anthropometric measurements, most newborns were AGA (weight AGA 74.8%, length AGA 86.4%, head circumference AGA 86.0%).

Weight, length, and head circumference at birth were 3323.2 ± 495 g, 49.7 ± 2.4 cm, and 34.1 ± 2.7 cm, respectively.

We did not find any significant association between ethnicity, peripartum, and newborn characteristics or complications.

Multivariable regression models were used to test predictors of newborns' anthropometric parameters. The multilinear model for each anthropometric parameter included age at diagnosis, BMI at the time of the first visit, geographic origin, GDM in previous pregnancies, gestational age, and type of treatment (diet as reference).

Newborn weight was independently predicted by gestational age at delivery (*B* = 177.176, CI 95% 136.030–218.322, *p* < 0.0001) and GDM in previous pregnancies (*B* = 106.382, CI 95% 5.732–207.032, *p*=0.04), newborn height by gestational age at delivery (*B* = 0.791, CI 95% 0.584–0.998, *p* < 0.0001), and insulin treatment (*B* = 0.692, CI 95% 0.046–1.338, *p*=0.03), whereas head circumference only by gestational age at delivery (*B* = 0.269, CI 95% 0.088–0.432, *p*=0.004).

## 4. Discussion

GDM is a major public health problem, affecting about one in every six pregnancies globally [15]. In the present study, we retrospectively investigated clinical characteristics and perinatal outcomes in a heterogeneous population of Caucasian women born in Italy and HMPC and affected by GDM.

First, we observed a different therapy load in HMPC women. In our population, HMPC women were younger, confirming findings of other studies conducted in Italy [16] and required more frequent insulin treatment than Caucasian women born in Italy. Over time, an exchange of habits between HPMC people and the host population has taken place. Regarding food habits, HPMC people have modified their dietary habits, influenced by the western food culture [17]. Ethnic minority groups have developed a higher risk than the host population of becoming obese and developing lifestyle-related diseases, such as T2DM and cardiovascular diseases (CVD) [18]. In this context, the insulin therapy suggestion following a worse glycemic control in HMPC pregnant women could be explained by a different ethnic background and inadequate food education with difficulties in understanding and following the dietary advice proposed by the medical staff. The recommended diet is based on Mediterranean eating habits, which could differ substantially from those of non-Western countries. Moreover, the transition to a western diet typical of the industrialized countries could influence BMI and poor glycemic control according to the defined theory of Nutrition Transition [19]. The Nutrition Transition is driven by industrialization and globalization of the food market. It has led to an increased supply of ultraprocessed foods, which are cheap and rich in fat, sugar, and other refined carbohydrates [20, 21]. The result has been increased intake of energy-dense foods and snacks, sugary drinks, and a shift toward more meat and dairy. Concomitantly, the intake of whole grains and legumes is reduced [20]. Moreover, urbanization has led to less physical activity and thus a lesser need for food energy. Another process to be considered in nutritional management is dietary acculturation that is defined as when a minority group adopts food choices of the host country, including changes in identity, attitudes, and values that accompany individual movement from their original culture toward the mainstream culture in the new country [22]. Studies demonstrated that HPMC people may find new ways to cook traditional dishes and meals, excluding typical foods, and consuming new foods, frequently unhealthy [23, 24]. Indeed, dietary changes in relation to migration are complex and dietary patterns should be further examined according to the length of residence of immigrant women in the host country [25, 26]. Thus, HPMC people may need more nutritional advice and treatment. On the contrary, health personnel feel often poorly equipped to educate these groups due to insufficient knowledge of native diet and new lifestyle habits that occurred after migration [27]. However, the prescription of diets that consider the country of origin of the patients, proposing well-known foods, resulted in greater adherence to the diet and better metabolic control in pregnant HPMC women, strengthening the role of education of both patients and healthcare providers [28].

Despite the previous argumentation, evidence in countries with a long migratory history showed that immigrants had comparable or better perinatal health outcomes than natives despite socioeconomic disadvantages [29]. These findings were confirmed by our results, showing no difference in terms of outcomes between ethnic groups. This observation is commonly described in the literature as the “healthy migrant paradox” [30] and contradicts the axiom that socioeconomic inequalities translate into health inequalities. In this context, interestingly, Juarez et al. [31] hypothesized that low birth weight and preterm deliveries due to standardized procedure of anticipating delivery in GDM patients could be at the origin of the healthy migrant paradox. In line with our results, a recent Italian study demonstrated that the interaction of GDM and ethnicity did not identify estimated risks of adverse outcome. Women with GDM might receive more attention from the national health system, thus reducing any potential disadvantage related to the status of migrants, supporting the healthy migrant paradox [32]. Conversely, in a Spanish study, clinical characteristics and perinatal outcomes of women with GDM were different according to ethnicity [33]. However, our population could not be matched with the Spanish one considering the high prevalence of Moroccan and Latin American women among the latter.

Second, higher fasting and 120-minute OGTT levels, as well as BMI, increased the risk of insulin treatment. Moreover, the presence of two or more diagnostic OGTT glucose levels doubled the risk of starting insulin therapy. Previous studies, although heterogeneous for screening methods, diagnostic criteria for GDM, blood glucose thresholds, glycemic control standards, and population characteristics, investigated the risk of insulin treatment in pregnant women with GDM [34–38]. The most recent study by Tang et al. [39] demonstrated that fasting glucose level, 1 h plasma glucose after OGTT, glycosylated hemoglobin, maternal age, pregestational and maximum weight, pregestational BMI, family history of diabetes in first-degree relatives, acanthosis nigricans, and prenatal weight were potential predictors of insulin treatment during pregnancy, confirming previous evidence. These insights are significant as patients should be stratified according to the personal risk of insulin treatment allowing tailored management for each subgroup. Thus, resources and intensity of care could be applied appropriately. Recently, a nomogram for the prediction of insulin requirement in a Chinese population with GDM has been developed [40]. The nomogram, which incorporated seven indicators (maternal age, gestational age at GDM diagnosis, BMI at GDM diagnosis, family history of T2DM in first-degree relative, history of GDM, fasting glucose level, and HbA1c), has been proposed to help in stratifying GDM women for a closer follow-up, driving in the allocation of medical resources according to personal risk factors.

Third, considering peripartum and newborn characteristics, we did not find any significant association between individual ethnicities and neonatal/maternal complications, as discussed above. Overall, newborn weight was independently predicted by gestational age at the delivery and GDM in previous pregnancies, newborn height by gestational age and insulin treatment, and head circumference by gestational age at delivery. In a recent large study (894 GDM women) conducted over 13 years in the north of Italy on a multiethnic population [16], macrosomia was associated with BMI and weight gain, but also with previous macrosomia or GDM, confirming our findings.

The present study has some limitations. First, we did not obtain data on other risk factors such as socioeconomic and education information and smoking status, which has been reported to influence perinatal outcome regardless of ethnicity. Furthermore, the prevalence of anamnestic characteristics as diabetes family history could be underestimated considering that the data is self-reported and health care information on first-degree relatives living in other countries could be missing. Furthermore, the length of living in Italy of HPMC women has not been evaluated. However, the homogeneity in GDM diagnostic criteria and delivery of care strengthens our findings.

In conclusion, in our multiethnic population of women affected by GDM, the need for intensive care and insulin treatment is high in HPMC women although the frequency of adverse peripartum and newborn outcomes did not vary among ethnic groups, despite the different ages. Thus, nutritional intervention and education should be targeted toward the prioritization of higher-risk groups and adaptation of the program to a multiethnic population. Characteristics of glucose response to OGTT and BMI are advantageous and cheap parameters to tailor the timing of follow-up.

## Figures and Tables

**Table 1:** Peripartum and newborn characteristics assessed in the study.

Variables	
Gestational age	Weeks

Type of delivery	Spontaneous vaginal delivery
	Vaginal delivery with forceps or suction cup
	Scheduled caesarean delivery
	Urgent caesarean delivery

Presentation	Vortex
	Podex
Dystocia	

Vitality	Born alive
	Stillborn

1 min APGAR	7–10
	4–6
	0–3

5 min APGAR	7–10
	4–6
	0–3
Meconium	
Malformations	

Weight	SGA
	AGA
	LGA

Height	SGA
	AGA
	LGA

Head circumference	SGA
	AGA
	LGA

**Table 2:** GDM risk factors and differences among groups according to the area of origin.

Variables	Total (*N* = 586)	Area of origin								
		Italy (*N* = 333)	East Europe (*N* = 52)	Central Asia (*N* = 86)	Cina and Mongolia (*N* = 13)	North Africa (*N* = 61)	Central Africa (*N* = 18)	South America (*N* = 19)	Other (*N* = 4)	*p* value
Age at pregnancy (years)	34.2 ± 5.0	**35.2 ± 4.6**	**32.9 ± 6.0** ^§^	**31.8** ± **5.0**^£^	**31.9** ± **3.7**^§^	**33.4 ± 4.9** ^§^	34.4 ± 5.3	34.9 ± 5.5	35.0 ± 5.8	**<0.0001**
BMI (kg/m^2^)	29.1 ± 5.5	**28.7 ± 5.9**	29.8 ± 5.2	29.3 ± 4.0	28.7 ± 4.5	29.4 ± 4.4	**32.0 ± 6.4** ^*∗*^	**32.0 ± 6.9** ^*∗*^	25.2 ± 1.0	0.03
No. of pregnancies	2.1 ± 1.1	**1.8 ± 0.8**	**2.2** ± **1.1**^§^	**2.4** ± **1.2**^£^	**2.5** ± **1.3**^§^	**2.7** ± **1.3**^£^	**2.2** ± **0.9**^*∗*^	**2.5** ± **1.2**^§^	2.8 ± 2.1	**<0.0001**
Previous GDM	120/386 (31.1%)	58/195 (29.7%)	10/35 (28.6%)	19/62 (30.6%)	4/10 (40.0%)	20/52 (38.5%)	2/14 (14.3%)	7/16 (43.8%)	0/2	0.54
Miscarriage	85 (14.5%)	55 (16.5%)	8 (15.4%)	8 (9.3%)	1 (7.7%)	7 (11.5%)	2 (11.1%)	3 (15.8%)	1	0.66
Family history for GDM	354 (60.4%)	**214 (64.3%)**	**26 (50.0%)** ^*∗*^	48 (55.8%)	6 (46.2%)	42 (68.9%)	**5 (27.8%)** ^§^	12 (63.2%)	1	**0.01**

^∗^ *p* < 0.05, ^§^*p* < 0.01, ^&^*p* < 0.001, and ^£^*p* < 0.0001. Significant differences are shown in bold.

**Table 3:** Gestational metabolic characteristics and differences among groups according to the area of origin.

Variables		Total (*N* = 586)	Area of origin								
			Italy (*N* = 333)	East Europe (*N* = 52)	Central Asia (*N* = 86)	Cina and Mongolia (*N* = 13)	North Africa (*N* = 61)	Central Africa (*N* = 18)	South America (*N* = 19)	Other (*N* = 4)	*p* value
Week of pregnancy		26.2 ± 5.4	26.3 ± 4.9	27.2 ± 5.5	25.9 ± 6.1	28.2 ± 4.3	25.3 ± 6.1	24.8 ± 6.8	26.1 ± 7.0	27.8 ± 2.2	0.34
2 h OGTT glucose level (mg/dL) *Data available for 337 pt*	T 0′	94.7 ± 15.3	93.1 ± 18.7	91.8 ± 6.6	97.2 ± 12.1	93.8 ± 9.8	98.8 ± 9.9	99.8 ± 8.6	96.4 ± 11.6	84.0 ± 12.7	0.17
	**T 60′**	169.5 ± 36.9	**170.8** ± **34.9**	179.3 ± 41.4	**160.1** ± **36.8**^*∗*^	151.8 ± 41.4	177.4 ± 35.8	**150.5** ± **41.1**^*∗*^	183.7 ± 37.6	173.0 ± 38.2	0.02
	T 120′	142.1 ± 32.2	142.7 ± 31.3	137.3 ± 31.2	137.1 ± 33.8	159.2 ± 34.5	146.2 ± 31.7	142.8 ± 42.0	156.7 ± 24.4	129.0 ± 42.4	0.35

2 h OGTT diagnostic time *Data available for 337 pt*	T 0′	115 (34.1%)	54 (31.8%)	11 (34.4%)	28 (41.8%)	1 (20.0%)	12 (32.4%)	7 (53.8%)	1 (9.1%)	1	0.31
	T 60′	45 (13.4%)	27 (15.9%)	8 (25.0%)	7 (10.4%)	0 (0.0%)	2 (5.4%)	0 (0, 0%)	1 (9.1%)	0	0.16
	**T 120′**	35 (10.4%)	**25 (14.7%)**	1 (3.1%)	5 (7.5%)	2 (40.0%)	**0 (0.0%)** ^§^	1 (7.7%)	1 (9.1%)	0	0.03
	More	142 (42.1%)	**64 (37.6%)**	12 (37.5%)	27 (40.3%)	2 (40.0%)	**23 (62.2%)** ^§^	5 (38.5%)	**8 (72.7%)** ^*∗*^	1	0.09
Treatment	Diet	**465 (79.4%)**	**274 (82.3%)**	**6 (11.5%)** ^£^	65 (75.6%)	**7 (53.8%)** ^§^	**21 (34.4%)** ^£^	15 (83.3%)	**5 (26.3%)** ^£^	4	**0.007**
	Insulin	**121 (20.6%)**	**59 (17.7%)**	**46 (88.5%)** ^£^	21 (24.4%)	**6 (46.2%)** ^§^	**40 (65.6%)** ^£^	3 (16.7%)	**14 (73.7%)** ^£^	0	

^∗^ *p* < 0.05, ^§^*p* < 0.01, ^&^*p* < 0.001, and ^£^*p* < 0.0001. Significant differences are shown in bold.

**Table 4:** Peripartum and newborn characteristics and differences among groups according to the area of origin.

Variables		Total (*N* = 586)	Area of origin								
			Italy (*N* = 333)	East Europe (*N* = 52)	Central Asia (*N* = 86)	Cina and Mongolia (*N* = 13)	North Africa (*N* = 61)	Central Africa (*N* = 18)	South America (*N* = 19)	Other (*N* = 4)	*p* value
Gestational age at delivery (weeks)		38.4 ± 1.4	38.4 ± 1.5	38.7 ± 1.7	38.4 ± 1.3	38.0 ± 2.5	38.5 ± 1.4	38.2 ± 1.0	38.2 ± 1.2	38.0 ± 1.4	0.84

Type of delivery *Data available for 331 pt*	Spontaneous vaginal delivery	213 (64.4%)	**106 (59.9%)**	21 (67.7%)	37 (71.1%)	3 (60.0%)	**29 (78.4%)** ^*∗*^	7 (58.3%)	8 (57.2%)	2	0.37
	Vaginal delivery with forceps or suction cup	10 (3.0%)	5 (2.8%)	1 (3.2%)	3 (5.8%)	0 (0.0%)	0 (0.0%)	0 (0.0%)	0 (0.0%)	0	0.71
	Scheduled caesarean delivery	57 (17.2%)	**35 (19.8%)**	6 (19.4%)	**4 (7.7%)** ^*∗*^	0 (0.0%)	4 (10.8%)	3 (25.0%)	5 (35.7%)	0	0.12
	Urgent caesarean delivery	51 (15.4%)	31 (17.5%)	3 (9.7%)	8 (15.4%)	2 (40.0%)	4 (10.8%)	2 (16.7%)	1 (7.1%)	0	0.54

Presentation *Data available for 331 pt*	Vertex	310 (93.7%)	164 (92.7%)	30 (96.8%)	49 (92.5%)	4 (80.0%)	36 (97.3%)	13 (100%)	13 (92.9%)	2	0.65
	Podex	21 (6.3%)	13 (7.3%)	1 (3.2%)	4 (7.5%)	1 (20.0%)	1 (2.7%)	0 (0.0%)	1 (7.1%)	0	

Dystocia *Data available for 331 pt*		2 (0.6%)	**0 (0.0%)**	0 (0.0%)	**2 (3.8%)** ^§^	0 (0.0%)	0 (0.0%)	0 (0.0%)	0 (0.0%)	0	0.11

Vitality *Data available for 331 pt*	Born alive	329 (99.7%)	177 (100%)	31 (100%)	52 (98.1%)	5 (100%)	36 (100%)	12 (100%)	14 (100%)	2	0.52
	Stillborn	1 (0.3%)	0 (0.0%)	0 (0.0%)	1 (1.9%)	0 (0.0%)	0 (0.0%)	0 (0.0%)	0 (0.0%)	0	

1 min APGAR *Data available for 329 pt*	**7–10**	305 (92.7%)	**166 (93.8%)**	**25 (80.7%)** ^*∗*^	47 (90.4%)	5 (100%)	34 (94.4%)	12 (100%)	14 (100%)	2	0.13
	**4–6**	16 (4.9%)	**7 (3.9%)**	**5 (16.1%)** ^§^	2 (3.8%)	0 (0.0%)	2 (5.6%)	0 (0.0%)	0 (0.0%)	0	0.11
	0–3	8 (2.4%)	4 (2.3%)	1 (3.2%)	3 (5.8%)	0 (0.0%)	0 (0.0%)	0 (0.0%)	0 (0.0%)	0	0.65

5 min APGAR *Data available for 329 pt*	7–10	323 (98.2%)	174 (98.3%)	30 (96.8%)	50 (96.2%)	5 (100%)	36 (0.0%)	12 (100%)	14 (100%)	2	0.84
	4–6	5 (1.5%)	2 (1.1%)	1 (3.2%)	2 (3.8%)	0 (0.0%)	0 (0.0%)	0 (0.0%)	0 (0.0%)	0	0.72
	0–3	1 (0.3%)	1 (0.6%)	0 (0.0%)	0 (0.0%)	0 (0.0%)	0 (0.0%)	0 (0.0%)	0 (0.0%)	0	0.99

Meconium *Data available for 330 pt*		69 (20.9%)	37 (20.9%)	7 (22.6%)	12 (22.6%)	2 (40.0%)	5 (13.9%)	2 (16.7%)	4 (28.6%)	0	0.82

Malformations *Data available for 330 pt*		4 (1.2%)	1 (0.6%)	1 (3.2%)	1 (1.9%)	0 (0.0%)	1 (2.8%)	0 (0.0%)	0 (0.0%)	0	0.82

^*∗*^ *p* < 0.05, ^§^*p* < 0.01, ^&^*p* < 0.001, and ^£^*p* < 0.0001. Significant differences are shown in bold.

## Data Availability

Data repository is available upon request to the corresponding author.
